# A German-language replication study analysing the role of figurative speech in reasoning

**DOI:** 10.1038/sdata.2016.98

**Published:** 2016-10-25

**Authors:** Ursula Christmann, Anne-Louise Göhring

**Affiliations:** 1 Professor of Psychology at the (Ruprecht-Karls-)University of Heidelberg, 69117 Heidelberg, Germany; 2 M.Sc. Graduate student in Psychology at the (Ruprecht-Karls-)University of Heidelberg, 69117 Heidelberg, Germany

**Keywords:** Human behaviour, Communication

## Abstract

According to the metaphorical framing model, the use of metaphors in discussing an issue influences recipients’ understanding and assessment of that issue. In a recent study, participants read a text referring to a city’s crime problem either as a *beast* or a *virus* and then proposed counter-measures for that problem. Participants’ suggestions differed depending on the metaphor they had read. This replication matched the original procedure regarding the content of the rhetorical figures (*beast* vs *virus*), the topic under focus (crime) and the measurement of the dependent variable (open-end format to collect participants’ proposals). The procedure differed from the original with respect to language (German instead of English) and by including the formal type of rhetorical figure (metaphor or simile) as a factor. A systematic influence of the content of the figure on subjects’ proposals was observed. Presenting the rhetorical figure as a metaphor or a simile had no effect. Taken together, we were able to replicate the main effect of the original study. Metaphors do indeed frame reasoning.

## Background & Summary

The research behind this study builds on the idea that using metaphors to talk about an issue is not merely a flowery way of expressing an abstract concept but in fact represents and structures reasoning about that concept itself^1^. The metaphor operates as a frame that guides interpretation, a phenomenon Robins^2^ has called the metaphor framing effect with reference to Lakoff and Johnson’s *Metaphors We Live By* ^3^. The phenomenon is especially widespread in the domain of socio-political discourse. For example, in Europe the refugee problem is commonly referred to as the ‘refugee flood’, a frame suggesting an analogy between the refugee issue itself and the concept of a flood that needs to be stemmed, e.g., by building dams. A frame-consistent option for action would be to put up fences to stop refugees. Similarly, after the September 11 attacks, the United States declared ‘war on terror’, which in turn suggested justification for military action. These examples establish a frame that encourages evaluative conclusions about situations or issues and guides potential lines of thought and action in a very specific way. There are empirical findings from a variety of contexts indicating that the use of rhetorical figures can be a powerful tool for influencing people (e.g., in politics, health matters, advertisements, etc. refs 4–7).

One widely acknowledged study on the subject was recently conducted by Thibodeau and Boroditsky^8^. It investigates the effect of metaphors in connection with a socio-political issue, namely crime. Participants were given a brief text discussing the crime problem in a fictitious city. One version of the text began with ‘Crime is a virus infecting the city of Addison’, the other version with ‘Crime is a wild beast preying on the city of Addison’^8^. The metaphor, *virus* or *beast*, was taken up once more later in the text. Apart from that, the texts were identical. Participants were given one version of the text, asked to read it and then to suggest measures that the city should take to deal with the problem. The authors’ hypothesis was that the measures suggested by the participants would differ depending on which of the metaphors was present in the text. They expected that in the *virus* version participants would be likely to think about crime in terms of a contagious disease. Accordingly, they would tend to recommend what the authors called ‘reform’ measures, i.e., measures aimed at diagnosing the origin of the *virus*, treating it and preventing contagion. On the other hand, participants reading the *beast* version were expected to conceptualize the crime problem in terms of an attack by a wild animal. Here it was assumed that the participants would suggest measures—referred to by the authors as ‘enforcement’ measures—aimed at taking criminals into custody, the enforcement of existing laws and the punishment of offenders.

In fact, this prediction turned out to be accurate. A majority of participants proposed measures that were compatible with the metaphor used in the text. Obviously, the metaphor introduces distinctive perspectives on the same issue and—more importantly—activates different options for action. The basic idea informing this experiment was confirmed by the authors on various occasions and in a number of variations^8^. For example, the effect persisted when the metaphorical frame was established by one single word only, whereas it vanished when instead of the metaphors in the text the words *virus* or *beast* were only presented as a prime before a neutral version of the text.

On the other hand, when the main experiment by from Thibodeau and Boroditsky^8,9^ was replicated by Steen, Reijnierse and Burgers,^10^ their findings did not confirm the original result. Thereupon, Thibodeau and Boroditsky^11^ adjusted the norms for answer-coding, reanalysed the data provided by Steen *et al.* ^10^ and thus replicated the effect after all. In short, despite the convincing initial evidence, it was impossible to draw any clear-cut conclusions. For this reason, a replication of the central experiment was conducted in German. The objective was to replicate the metaphor-framing effect empirically confirmed by Thibodeau and Boroditsky^8,9,11^. We focused on the first and central experiment, just as Steen *et al.* ^10^ had done. A translation of the original material was used, so our procedure matched the original with respect to topic (crime), metaphors (*virus*, *beast*) and response format (open-end).

## Method

The investigation was conducted using a 2×2 between-subjects design. The first factor had two levels and related to the content of the metaphor, *beast* vs *virus*. As a precaution, and unlike Thibodeau and Boroditsky^8^, we added a second factor also operative on two levels that referred to the type of figure used: metaphor (*crime is a virus/beast*) vs simile (*crime is like a virus/beast*). The *beast* metaphor is rather uncommon in German. There is evidence that aptness or ‘fit’ plays an important role in understanding metaphors. Glucksberg^12^ summarises his findings to the effect that common metaphors are automatically processed much faster than similes by accessing an abstract metaphor category and applying all the attributes of the abstract category to the target object. Uncommon metaphors, on the other hand, are processed as if they were similes, i.e., by matching attributes of the simile’s source domain to the simile’s target object one by one.

As in Thibodeau and Boroditsky’s study^8^, the dependent variable was the type of measure suggested by the participants to deal with the crime problem. As a control variable, participants were also asked to indicate which passage in the text had been most influential in prompting their suggestions. The investigation was conducted as a paper-and-pencil study.

### Sample

In the original study^8^, data were collected from a rather large sample (*N*=485). While it is of course desirable to avoid a sample that is too small, having a very large sample size can also be problematic. If samples are very large, purely coincidental differences may be identified as significant. Accordingly, we ran an estimation of the sample size required using G*Power 3.1.9.2 software. This was done assuming a medium-sized effect of *w*=0.3 and using the conventional values of 0.05 for the significance level and.80 for power desired. The degrees of freedom were computed using the formula (*k*-1)*(*p*-1), where *k* and *p* are the number of lines and columns in the contingency table. This estimation resulted in a required sample size of *N*=88, which corresponds to a minimum of 22 participants for each of the four cells.

Altogether, 122 individuals took part in the study. The data from 7 of the participants had to be excluded from the analysis (see section Exclusion of cases), so the final sample consisted of 115 individuals (83 females, 32 males). Their ages ranged from 18 to 51 years with a mean of 22.4 and a standard deviation of 3.9. Participants were semi-randomly assigned to one of four experimental groups of equal size (28 for the *beast* simile and 29 each for the *virus* metaphor, the *virus* simile and the *beast* metaphor). All participants were native German speakers and university students or graduates. 42.6 percent of the participants were majoring in psychology, 19.2 percent in politics and 30.4 percent in other subjects; 9.8 percent did not indicate their subjects. Participation was voluntary; participants could choose between attending the experiment to fulfil a course requirement or receiving a small reimbursement.

### Data and materials

The independent variables *content* and *type of figure*, the dependent variable *suggested measures* and the control variable *most influential passage* featured a nominal level of measurement.

#### Materials

The experimental materials were an introduction and a short text identical to the one used by Thibodeau and Boroditsky^8^. This text, which had been translated into German, was a short report on the crime problem of the fictitious city of ‘Addison’ (six lines, 69–71 words). In accordance with the two-by-two design, four versions of this text were generated. They contained the *virus* or the *beast* figure either as a metaphor or as a simile: 1. *virus* metaphor; 2. *beast* metaphor; 3. *virus* simile; 4. *beast* simile. As in the original study, the content of the figure (*virus/beast*) was taken up once more in the text to strengthen its effect. To this end, a description in line with the linguistic figure was chosen (*virus*: ‘it seems that crime is plaguing every neighbourhood’; *beast*: ‘it seems that crime is lurking in every neighbourhood’). Apart from the content of the respective figure and this one back-up, the four text versions were identical. The versions with the *virus* or the *beast* metaphor are shown below in English. The complete German material can be found in the data repository (study materials, Data Citation 1).

‘Crime is a {wild beast preying on/virus infecting} the city of Addison. The crime rate in the once peaceful city has steadily increased over the past three years. In fact, these days it seems that crime is {lurking in/plaguing} every neighborhood. In 2004, 46,177 crimes were reported compared to more than 55,000 reported in 2007. The rise in violent crime is particularly alarming. In 2004, there were 330 murders in the city, in 2007, there were over 500^8^.’

#### Measurement of the dependent variable and the control variable

Participants’ suggestions for measures to reduce crime were obtained by asking them: ‘In your opinion, what does Addison need to do to reduce crime?’^8^. Eleven lines were provided for the answers. Participants were also asked to underline the part of the report that had been most influential for their decision. This information was measured as a potential control variable. Participants’ answers are presented in the data repository (participants’ answers, Data Citation 1).

#### Answer coding

To code the measures suggested by the participants in the open-end question we established a coding system on the same lines as the one used by Thibodeau and Boroditsky^8^. This system had two main categories: ‘initiate or strengthen reforms’ and ‘enforce or intensify existing (precautionary) measures’. The corresponding categories in Thibodeau and Boroditsky^8^ are ‘reform’ and ‘enforce’ and were coded as 1 and 2 respectively. For both of these main categories we specified three subcategories that were mainly explicitations of the aspects referred to by Thibodeau and Boroditsky^8^. For the main category ‘initiate or strengthen reforms’ these were ‘analysing causes of the problem’, ‘eliminating the causes’, and ‘prevention: measures that are taken before the problem occurs’. Similarly, the main category ‘enforce or intensify existing (precautionary) measures’ included the aspects ‘concrete measures to take suspicious individuals in custody’, ‘control measures’, and ‘threat and imposition of more severe punishment’. The table used for coding participants’ answers is given in the data repository (table used for answer coding, Data Citation 1).

Coding itself was done largely in accordance with Thibodeau and Boroditsky^8^ and compliance with the following rules: If a subcategory was mentioned in the answer, one point was given for that subcategory. The maximum score for each subcategory was one point. The answer as a whole was coded as belonging to the main category (1 or 2) of which more aspects were included. If there were an equal number of aspects from both main categories, the answer was coded as 0 (‘both’). In this regard, our coding differed from Thibodeau and Boroditsky’s procedure^8^, where, in such a case, the point for the answer was split in half and each of the main categories’ frequencies incremented by 0.5. Part (26 percent) of the answers were coded by two raters with a Cohens κ=0.85, indicating very high interrater reliability. All disagreements between the coders were resolved before data analysis.

### Procedure

After the texts had been translated and adapted, there were four versions of the study material differing in terms of the text version they contained. Assigning versions to participants was done in a semi-randomised manner, i.e., the sets of study material were alternately sorted and handed out to participants in that order.

The material began by welcoming and thanking the participants. After that, participants were asked to read the text about the city’s crime problem and to answer the open-end question about what they thought the city should do to solve the problem. Furthermore, they were asked to indicate the passage of the text that had been most influential for their decision. Finally, the participants were asked to supply some demographic information regarding age, sex, education and subject studied. Data analysis was done using IBM SPSS Statistics versions 22 and 20.

## Data Records

The data records discussed in this section are available from the Open Science Framework (OSF). Data Citation 1 comprises one pdf file (study materials, participants’ answers, table used for answer coding, Data Citation 1) and one Excel file (coded responses, Data Citation 1).

The first part of the pdf file contains the entire study materials in the original German version. The second part of the pdf file takes the form of a table containing the following information: number of participant, experimental condition the participant was in, transcribed answer in its original form, demographics (age, gender, education). Participation was voluntary. Subjects were assured that their data would be treated anonymously and that it would be impossible to infer their individual identity. The table used for answer coding is available in the third part of the pdf file and is also presented in German. The Excel file shows the coded responses of the participants.

## Technical Validation

In line with Thibodeau and Boroditsky^8^, chi-square tests were performed for the dependent-variable type of suggested measure. All analyses refer to a level of significance of five percent.

### Exclusion of cases

Seven of the 122 responses (5.7%) did not fit into either category because the response lacked a concrete suggestion for reducing crime (e.g., ‘I need more information’, ‘the text doesn’t say’) and could therefore not be coded. These data were omitted from analysis. Accordingly, the final sample was made up of 115 individuals.

### Descriptive statistics

Overall, there was a pronounced tendency among the participants to suggest measures assignable to the category ‘initiate or strengthen reforms’. 48.7 percent of the answers belonged to this category, whereas 25.2 percent matched the category ‘enforce or intensify existing (precautionary) measures’ and 26.1 percent contained an equal number of aspects from both categories and were therefore coded as ‘both’ (χ^2^(2)=12.23, *P*=0.002). Considering only those answers that clearly matched one of the two main categories, 65.9 percent belonged to the category ‘initiate or strengthen reforms’ and 34.1 percent belonged to the category ‘enforce or intensify existing (precautionary) measures’ (χ^2^(1)=8.58, *P*=0.003). In Table 1 the absolute frequencies for all response categories are shown separately for the different versions (*virus, beast).*

Male and female participants were not equally distributed among the different groups. Firstly, there were more female than male participants overall (χ^2^(1)=22.62, *P*<0.001); secondly, this was also the case for three of the four versions (*virus* metaphor χ^2^(1)=4.17, *P*<0.05; *beast* metaphor χ^2^(1)=2.29, *P*=0.131; *virus* simile χ^2^(1)=4.17, *P*<0.05; *beast* simile χ^2^(1)=15.21, *P*<0.001). This unequal allocation is relatively unproblematic because gender was not associated with any specific response category in any of the four conditions (*virus* metaphor χ^2^(2)=4.29, *P*=0.177; *beast* metaphor χ^2^(2)=2.52, *P*=0.284; *virus* simile χ^2^(2)=2.66, *P*=0.264; *beast* simile χ^2^(2)<1).

### Data analysis compared to the original procedure

In contrast to Thibodeau and Boroditsky’s findings^8^, the current sample contained a substantial amount of answers that could not be clearly assigned to one of main categories and were thus coded as ‘both’. Thibodeau and Boroditsky^8^ handled these cases by sharing the point between the main categories and incrementing the frequencies of each of them by 0.5. Since this category was numerically strong and an analogous procedure would have seriously fudged the differences between the categories, the following analyses only take note of the responses that were unambiguously identified as belonging to one of the two categories.

### Influence of type of figure: metaphor vs simile

No overall difference was made in participant response frequencies by presenting the figure as a metaphor or a simile (χ^2^(1)<1). Additionally, the content of the linguistic figure was considered: again, no difference emerged in the group that was presented with the simile version of the *virus* or *beast* figure (χ2(1)<1). However, participants confronted with the metaphor version of the figure gave significantly more responses belonging to the category ‘enforce or intensify existing (precautionary) measures’ when crime was framed as a *beast* instead of a *virus* (χ^2^(1)=3.23, *P*=0.036, one-tailed testing).

### Correspondence of present data with original data

Since there was no overall difference between the two types of figure, there was no reason to treat the data separately with regard to this factor, so the data from the two levels were analysed conjointly.

**Figure 1:**
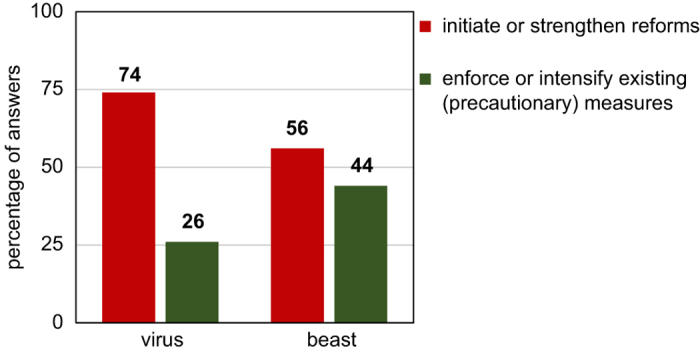
Depiction of the proportion of answers belonging to the two categories separately for the two conditions in percent.

Although there was a pronounced overall tendency to suggest measures aimed at preventing crime, the effect of the figures employed—*virus* vs *beast*—was largely as expected. Individuals in the *beast* groups were more likely to propose measures fitting the category ‘enforce or intensify existing (precautionary) measures’ than individuals in the *virus* groups (χ^2^(1)=3.33, *P*=0.034, one-tailed testing). The majority of the participants identified the statistics in the crime report or the information that crime had risen as having had the greatest effect on their decisions. Only 6 participants (7.1%) indicated that the linguistic figure had been the most influential passage of the text for their response. Excluding these participants from the analysis had no effect on the result when comparing answer frequencies for the two categories between the two conditions (χ^2^(1)=3.23, *P*=0.036, one-tailed testing). Nor was there any difference between the proportion of responses consistent with the figure between the two analyses (χ^2^(1)<1). Figure 1 indicates the percentage of answers belonging to the two categories.

Overall, our results represent a successful replication of the metaphor-framing effect reported by Thibodeau and Boroditsky^8^. When the *beast* metaphor was used, participants significantly more often proposed measures aimed at enforcement or intensification of existing (precautionary) measures. When including the proposals that could not clearly be assigned to one of the two main response categories (i.e., conducting the analysis in full accordance with the original procedure) this difference was not statistically meaningful. Most participants did not refer to the figure of speech as being crucial for their proposals. But eliminating those participants who did identify the linguistic figure did not change the results. The ‘type of figure’ factor—implemented as a precaution—did not affect the overall response pattern. Moreover, a pronounced tendency was discernible to favour measures for initiating or strengthening reforms. In this respect, our results differ from Thibodeau and Boroditsky’s^8^, where the opposite tendency was observed.

## Usage Notes

The objective of the present study was to replicate the metaphor-framing effect demonstrated by the first experiment conducted by Thibodeau and Boroditsky^8^. We observed that the metaphor used to refer to a city’s crime problem—*virus* or *beast*—had a systematic effect on the type of counter-measure proposed by the participants. Although participants generally favoured reform over enforcement, this trend was not observed in the group presented with the *beast* metaphor, which framed crime as an attack by a wild animal. Within the *virus* groups the tendency to favour reform and hence—in metaphorical terms—prevent contagion and encourage treatment was very pronounced. Thus, our results both confirm the findings of Thibodeau and Boroditsky^8^ and at the same time demonstrate that metaphor influences the way in which people think about an issue, making it a powerful persuasion device.

However, there were also some divergences between our results and the original study. The overall tendency to predominantly propose reform measures distinguishes the present study from Thibodeau and Boroditsky^8^, where the participants favoured enforcement measures. This may be due to differences in the samples. Although both samples were made up of university students, a group that tends to be politically more liberal than the overall population, culture-specific differences may exist between the American sample Thibodeau and Boroditsky^8^ used and our German sample with regard to crime-fighting policies and the role of government in such issues. In other variations of the experiment using a more representative sample, Thibodeau and Boroditsky^8^ found the same tendency for participants to favour enforcement, as did Steen *et al.*,^10^ who used one group of Dutch participants and three American samples. Other potential reasons for this pattern are different crime rates in the United States and Germany and different attitudes towards the ownership and use of guns in the two countries.

An additional ‘type of linguistic figure’ factor was introduced to detect potential differences in aptness between the two figures *virus* and *beast*. Although presenting the linguistic figure as a simile or a metaphor had no effect, some concerns regarding the *beast* metaphor in German remained. Firstly, compared to the word *virus,* the word *beast* is relatively rare. Secondly, as a metaphor it is mostly used to refer to a person, not situations or socio-political issues. *Virus*, on the other hand, is a very commonly used word in general and, as a metaphor, seems to be readily applicable to the generation and evolution of socio-political problems. It is possible that the overall tendency in favour of proposing reform measures may have partly originated from a perception of the *beast* metaphor as not being particularly apt. A small-scale post-experimental investigation (*N*=30 university students) substantiates this assumption. Subjects judged the *virus* metaphor to be more common and more apt than the *beast* metaphor. This implies that the *virus* metaphor but not the *beast* metaphor may have been sufficiently apt to actually fulfil the function of a frame. Of course, this cannot be safely concluded without some sort of neutral condition. In future research, a baseline condition should be implemented and the aptness of the respective metaphors should be examined separately.

In coding the data, there were a large number of responses that could not be assigned to either of the two main categories. In the original study^8^, this was not reported to have been a problem. And in the replication by Steen *et al.*,^10^ this problem could not arise because they did not use an open-end format. In that respect, our procedure differed from the original approach by excluding these answers from the analysis. This is a risk that comes with the use of an open response format along with a poorly differentiated category system. Using an open end format is, however, an ecologically valid way of measuring the metaphor-framing effect, because this effect is based on the selective salience of certain aspects of an issue that are enforced by the respective metaphor. Offering participants possible answers in a closed format, for example by having them rank or rate measures according to their suitability, would certainly be a more reliable method. It does not involve the potential hazards of ex-post response coding, but it is also less ecologically valid. There are two possible approaches to this problem for future investigations. Firstly, a more sophisticated coding system should be devised that is better able to cover the variability of subjects’ answers. Secondly, one could—and to our minds should—conduct a separate investigation comparing the two data-collection formats i.e., open-end vs closed.

Also, there may be potentially relevant moderators not considered in this study. For example, there is evidence that the framing effect is attenuated when knowledge about the topic under discussion is high^2^. Along these lines, Göhring and Christmann^13^ recently found that the use of metaphors in referring to current political issues in Germany (the refugee crisis, radical Islamism, the events in Cologne on New Year’s Eve) only influenced cognitive reasoning (attitudes and evaluations of measures) on these topics if subjects had little prior knowledge of them. And Thibodeau and Boroditsky^9^ have demonstrated that political affiliation and commitment influence the metaphor-framing effect. Subjects with strong political convictions seem to be more resistant to metaphor-framing on issues that do not tie in with their declared party programme. Therefore it is essential for future investigations to include measurements of relevant prior knowledge, and, where appropriate, of existing attitudes on the issue in question. Also, future samples should include a broader range of subjects in terms of age and education.

Altogether, the successful replication of Thibodeau and Boroditsky^8^ demonstrates that a metaphor-framing effect does exist and that it would be worthwhile investing more research effort in investigating the phenomenon in a differentiated manner and strengthening the evidence we already have.

Metaphors do influence our view of world. They provide a knowledge frame that guides the interpretation of complex subjects and situations, they make some aspects more salient and others less, they help to evaluate the information and define the scope for possible inferences and elaborations^5,9^. Thus they can be both used and abused as powerful cognitive tools conditioning attitude formation and attitude change vis-à-vis socio-political and cultural issues^4,14^. Accordingly, they are an ideal instrument for the mass media and as such, as Schwarz-Friesel^15^ has demonstrated in connection with metaphors on terrorism, can contribute to the collective awareness and memory of large sections of the population. At all events, we need to bear in mind that there are two sides to the same coin. Metaphors can be used in an emancipatory sense to heighten awareness of problems besetting vulnerable domains such as the environment (e.g., climate change) or migration policy. But they can also be used to obfuscate those same issues and manipulate the audience(s) exposed to them.

Quite apart from that, the question remains whether the (quasi-literary) framing via metaphor functions not only as a cognitive but also as an emotional tool in promulgating a particular view of the world. Investigating for this would surely represent a theoretical enhancement of the present metaphor-framing approach. It could for example be tested by comparing metaphor frames with argumentative frames in texts, drawing upon, say, ego-involvement as a potential moderator variable.

But metaphors are not only tools for persuasion and the generation of new meaning(s), they are also instruments of socio-political change because—as is amply demonstrated by the research on the metaphor-framing effect—they can guide the choice of options for decision-making and for action in many socially and economically relevant domains such as the environment, energy and health^6,7^. To fulfil this function optimally they need to have certain properties such as aptness, suitability and novelty^4,16^. In future studies on metaphor-framing, these qualities should be investigated more thoroughly and explicitly taken into consideration. Moreover, and especially with regard to the potential implementation of findings in applied research, we need to test whether the effect not only influences subjects’ thinking and reasoning but also their concrete actions in specific domains (e.g., donations or supportive behaviour). In this context it would, of course, also be of interest to extend one’s purview to the short- and long-term effects of metaphor frames on concrete action in comparison with purely cognitive (i.e., argumentative) frames. All these additional approaches could contribute much to the transfer of findings to communicative practice.

At all events, future studies should take into account a broader range of possible topics (politics, health, public life, economy, communication, etc.) and of possible metaphors^7^. The main focus should be placed on achieving a good match between metaphors and topics in the experimental material. The texts should use pretested, apt metaphors, avoid artificial constructions and be couched in a natural-sounding idiom in accordance with the standards of the participants’ mother tongue. Likewise, the costs and benefits of the response format should be reconsidered, perhaps trying out various ways of capturing the relevant effect. Last but not least, potential confounding factors, such as prior knowledge, attitude, age and level of education should be taken into account.

## Additional Information

**How to cite this article**: Christmann, U. & Göhring, A.-L. A German-language replication study analysing the role of figurative speech in reasoning. *Sci. Data* 3:160098 doi: 10.1038/sdata.2016.98 (2016).

**Publisher’s note**: Springer Nature remains neutral with regard to jurisdictional claims in published maps and institutional affiliations.

## Supplementary Material



## Figures and Tables

**Table 1:** Frequencies of the answer categories separately for the conditions (content of the metaphor) and overall.

**Answer categories**				
	**Initiate or strengthen reforms**	**Enforce or intensify existing (precautionary) measures**	**Both**	**Overall**
*Condition*				
*Virus*	31	10	17	58
*Beast*	25	19	13	57
Overall	56	29	30	115
